# Supplementary material for: Causal Relationship between Aging and Anorexia Nervosa: A White-Matter-Microstructure-Mediated Mendelian Randomization Analysis
Source: Biomedicines. 2024 Aug 16;12(8):1874. doi: 10.3390/biomedicines12081874 (PMC11351342; doi:10.3390/biomedicines12081874)
Supplement: Supplementary file 1 [file biomedicines-12-01874-s001.zip › Supplementary Figure/Figure S4/Singe SNP plot of MR analysis for TL to ubm-b-2020.pdf]

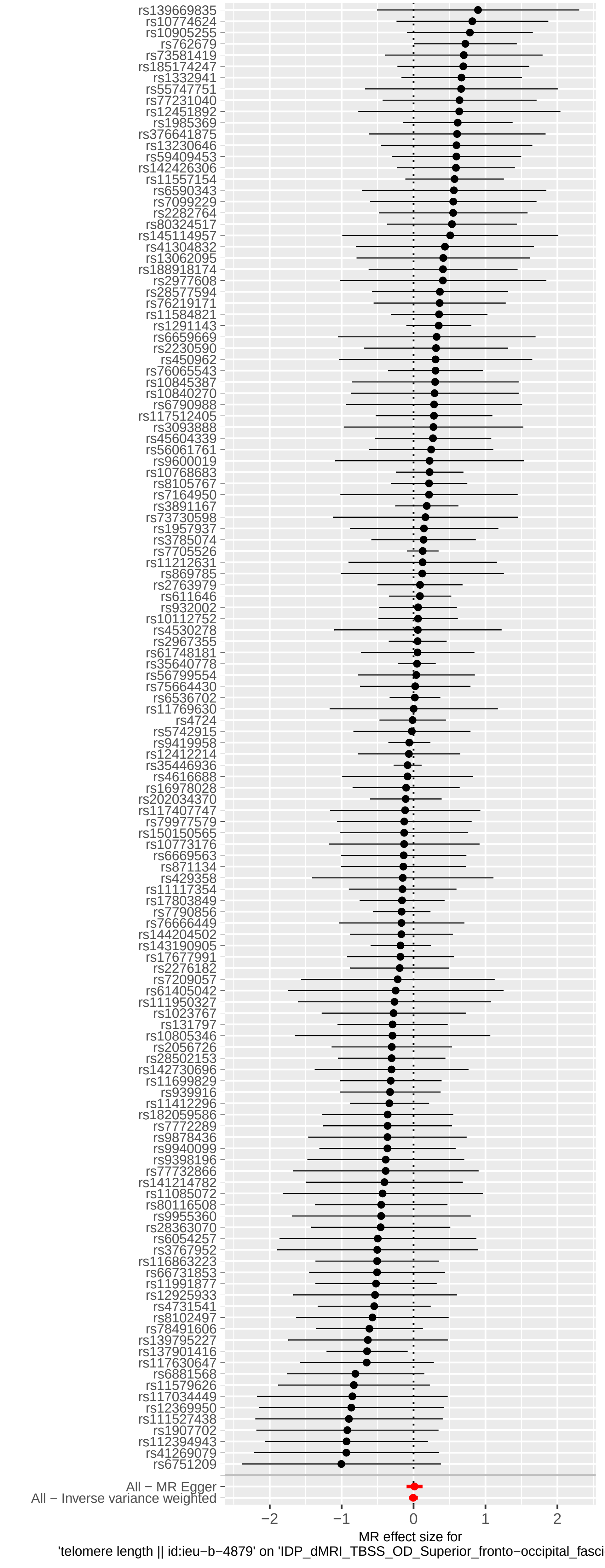
rs139669835
rs10774624
rs10905255
rs762679
rs73581419
rs185174247
rs1332941
rs55747751
rs77231040
rs12451892
rs1985369
rs376641875
rs13230646
rs59409453
rs142426306
rs11557154
rs6590343
rs7099229
rs2282764
rs80324517
rs145114957
rs41304832
rs13062095
rs188918174
rs2977608
rs28577594
rs76219171
rs11584821
rs1291143
rs6659669
rs2230590
rs450962
rs76065543
rs10845387
rs10840270
rs6790988
rs117512405
rs3093888
rs45604339
rs56061761
rs9600019
rs10768683
rs8105767
rs7164950
rs3891167
rs73730598
rs1957937
rs3785074
rs7705526
rs11212631
rs869785
rs2763979
rs611646
rs932002
rs10112752
rs4530278
rs2967355
rs61748181
rs35640778
rs56799554
rs75664430
rs6536702
rs11769630
rs4724
rs5742915
rs9419958
rs12412214
rs35446936
rs4616688
rs16978028
rs202034370
rs117407747
rs79977579
rs150150565
rs10773176
rs6669563
rs871134
rs429358
rs11117354
rs17803849
rs7790856
rs76666449
rs144204502
rs143190905
rs17677991
rs2276182
rs7209057
rs61405042
rs111950327
rs1023767
rs131797
rs10805346
rs2056726
rs28502153
rs142730696
rs11699829
rs939916
rs11412296
rs182059586
rs7772289
rs9878436
rs9940099
rs9398196
rs77732866
rs141214782
rs11085072
rs80116508
rs9955360
rs28363070
rs6054257
rs3767952
rs116863223
rs66731853
rs11991877
rs12925933
rs4731541
rs8102497
rs78491606
rs139795227
rs137901416
rs117630647
rs6881568
rs11579626
rs117034449
rs12369950
rs111527438
rs1907702
rs112394943
rs41269079
rs6751209
All − MR Egger
All − Inverse variance weighted
−2
−1
0
1
2
MR effect size for
'telomere length || id:ieu−b−4879' on 'IDP_dMRI_TBSS_OD_Superior_fronto−occipital_fasci
